# Genotypic distribution of HHV-8 in AIDS individuals without and with Kaposi sarcoma: Is genotype B associated with better prognosis of AIDS-KS?

**DOI:** 10.1097/MD.0000000000005291

**Published:** 2016-12-02

**Authors:** Tania Regina Tozetto-Mendoza, Karim Yaqub Ibrahim, Adriana Fumie Tateno, Cristiane Mendes de Oliveira, Laura Massami Sumita, Maria Carmem Arroyo Sanchez, Expedito José Luna, Ligia Camara Pierrotti, Jan Felix Drexler, Paulo Henrique Braz-Silva, Claudio Sérgio Pannuti, Camila Malta Romano

**Affiliations:** a Institute of Tropical Medicine Laboratory of Virology LIM52; b Department of Infectious Diseases, Clinics Hospital of the School of Medicine; c Institute of Tropical Medicine Laboratory of Seroepidemiology, University of São Paulo, São Paulo, Brazil; d Institute of Virology, University of Bonn Medical Centre, Bonn, German Centre for Infection Research (DZIF), partner site Bonn-Cologne, Germany; e Pathology Department of the School of Dentistry, University of São Paulo, São Paulo, Brazil.

**Keywords:** AIDS-KS, genotype, HHV-8, ORF K1, saliva

## Abstract

AIDS-associated Kaposi's sarcoma (AIDS-KS) caused by human herpes virus 8 (HHV-8) is the most severe and resistant form of KS tumor. Our aim was to verify whether there is an association between HHV-8 variability and development of AIDS-KS in Brazil by comparing the HHV-8 variability between individuals without and with KS. Saliva samples and blood, when available, were analyzed by polymerase chain reaction (PCR) techniques for detection of the fragments of ORF K1 of HHV-8, which were then genotyped and analyzed regarding the genetic variability. Our study described 106 positive cases for HHV-8 in the saliva from 751 AIDS patients without previous KS. In addition, we performed a phylogenetic analysis of HHV-8 in 34 of the 106 AIDS patients without KS and in 33 of the 37 patients with active KS. The distribution of HHV-8 genotypes A, B, C, and F in AIDS individuals was indistinguishable by comparing non-KS and KS groups, as well as regarding ethnicity. Considering the KS group, genotype B was associated with better prognosis of KS tumor. Interestingly, we found a particular profile of diversity within clade C and 2 recombinant patterns of HHV-8 in the saliva of AIDS individuals without KS. We emphasize the need to achieve standard genotyping protocol for ORF K1 amplification, thus allowing for substantial detection of HHV-8 variants. Our findings can shed light on the role of HHV-8 variability in the pathogenesis of AIDS-KS.

## 1 Introduction

HHV-8, a member of the gamma-herpesvirinae, is the etiologic agent of all clinical-epidemiological forms of Kaposi's sarcoma, namely, classic, endemic, iatrogenic and epidemic or AIDS-KS.^[1]^ Since the impact of HIV epidemic, AIDS-KS has been considered the most severe and treatment-resistant form of KS tumor.^[2,3]^ With despite the undetectable HIV viral load and recovery of CD4 levels following cART, a substantial proportion of persistent KS have been described in AIDS patients.^[4–6]^ Additional factors involved in the development and progression of AIDS-KS need to be further explored.

Although an association between HHV-8 variability and pathogenic potential has been demonstrated in some studies on classic, endemic and epidemic KS,^[7–11]^ other studies failed to do so.^[2,12,13]^ Such inconsistencies may be partially attributed to the lack of a standard method or protocol for HHV-8 genotyping, especially regarding the choice of targets in the HHV-8 genome and a more complete coverage of HHV-8 variants.

Investigation of biomarkers potentially useful for KS prognosis based on different loci of genome^[9,10,12,14,15,18]^ or whole-genome data^[16]^ has advanced parallel to the molecular characterization of a large number of HHV-8 strains infecting patients from different clinical and epidemiological forms of KS.^[9,10,12,14,15]^ For example, several short targets of HHV-8 from AIDS-KS lesions were selected to identify distinctive molecular pattern, but with restricted coverage of the repertoire of HHV-8 genotypes.^[17–19,12,20–23]^ Nowadays, sequence analysis of highly variable ORF K1 regions (i.e., VR1 and VR2) has allowed the identification of 7 major subtypes or genotypes of HHV-8 (A, B, C, D, E, F, and Z), all exhibiting clear ethnic and geographic clustering.^[10,12,15,21,23–27]^ In addition, the ORF-K1 encodes for a transmembrane signaling molecule that plays an important role in the lifecycle of HHV-8, provoking cellular activation, endothelial cell immortalization and works synergistically with HIV-1 Tat to promote tumors.^[28,29–30]^

There is scant information on the distribution of HHV-8 genotype in Brazil.^[13,18,21,23,25]^ Herein, we investigated whether there is an association between HHV-8 variability and development of AIDS-KS by performing a phylogenetic analysis of complete and partial K1 sequences of the HHV-8 genome and profiles of the viral genotype and subgenotype of AIDS individuals without or with KS.

## 2 Methods

### 2.1 Ethical issues and study groups

The present study protocol was approved by the Ethics and Research Committee of the University of São Paulo School of Medicine.

After the informed consent form was signed, samples of saliva or peripheral blood mononuclear cells (PBMCs) and serum were obtained from patients enrolled at the outpatient clinic of the HIV/AIDS Patient Care Extension Service (SEAP HIV/AIDS) of the Clinical Division of Infectious and Parasitic Diseases of the University of São Paulo School of Medicine Clinical Hospital (*ICHCFMUSP*), São Paulo, Brazil. All patients have been treated with cart under routine program.

Samples were collected from 2 different previous studies in 2 different situations that involved distinct groups. The first situation was characterized by the presence of HHV-8 among patients living with AIDS and without previous or concurrent KS manifestation. The period of HIV/AIDS diagnosis in this group was between 1987 and 2003. The second situation was characterized by the presence of AIDS-defining diagnosis of KS among patients who had anatomopathological and clinical diagnosis of active KS during the period from 1993 to 1998. Records on clinical progression of KS were based on medical consultations performed during 1998 and 1999, with a median of 4.41 consultations *per* patient. According to Krigel et al,^[31]^ the clinical stage of KS is defined as being of 3 possible clinical forms: visceral (VIS), localized cutaneous (CUT), and disseminated cutaneous (DISS). The clinical progression of KS was assessed based on the extension and number of KS lesions interpreted according to criteria set by Krown et al.^[32]^ Participants who had worsening of disease (e.g., lesion extension or new lesions) were grouped into subgroup W and those with no progression or improvement of KS were grouped into subgroup S.

#### 2.1.1 Non-KS group

In a previous cross-section study conducted from January 2007 through December 2008, saliva samples were collected from 751 individuals living with HIV/AIDS without previous KS. In the present study, we selected those samples with detectable HHV-8 DNA load to study the HHV-8 sequences.

In addition, we analyzed matched blood and saliva samples for 1 patient in this group taken at different times.

#### 2.1.2 KS group

In another previous cross-section study conducted from January 1998 through December 1999, PBMC samples were collected from 37 HIV/AIDS individuals with active KS. In the present study, we included these samples for comparative evaluation of HHV-8 sequences.

In addition, we investigated available serial blood samples or blood and saliva matched for these patients.

### 2.2 Samples and DNA extraction

#### 2.2.1 Non-KS group

Saliva was collected according to the protocol described by Beyari et al^[33]^ and cryopreserved at –70°C until analysis.

#### 2.2.2 KS group

The PBMC samples were obtained from 5 mL of total blood with EDTA by means of density gradient centrifugation using the Ficoll–Hypaque method (Sigma Chemicals, St. Louis, MO). A total of 1.0 × 10^6^ cells/mL were cryopreserved in liquid nitrogen at –192°C until analysis.

#### 2.2.3 DNA extraction

DNA was extracted from both KS and non-KS samples by using the Genomic DNA extraction kit (Real Genomics Real Biotech Corporation). DNA quality was monitored with PCR directed to beta-globin gene as internal control,^[34]^ whereas total DNA was quantified by using the NanoDrop spectrophotometer. All samples were suitable for viral DNA amplification. HHV-8 DNA was amplified with oligonucleotides directed to ORF-K1, recovering fragments from ∼300 to ∼860 bp,^[35,36]^ as shown in Table 1. A standard input of ∼200 ng of total DNA *per* reaction was used whenever possible. The input of samples with low concentrations was adjusted at the maximum volume of 10 μL.

**Table 1:**
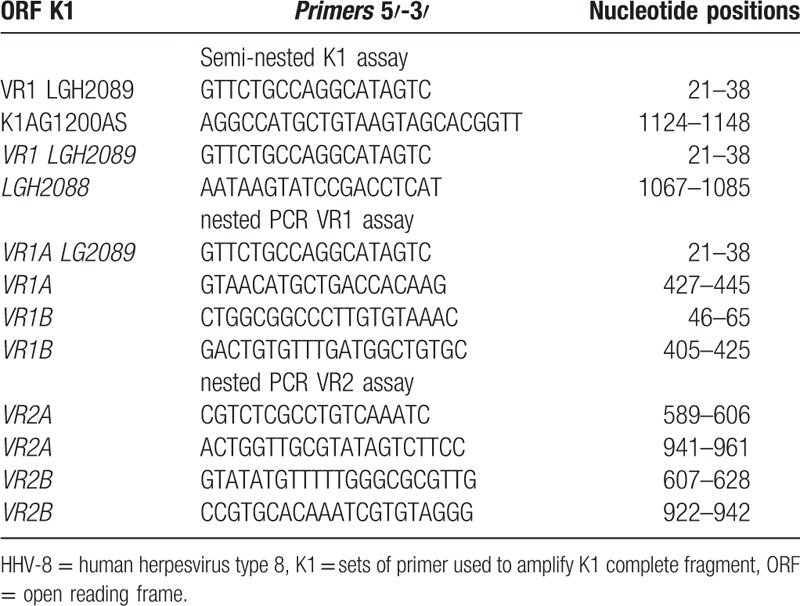
Primers used in complete and partial amplification of HHV-8 ORF-K1.

### 2.3 Detection of HHV-8 DNA in the saliva of Non-KS individuals

In order to compose a group of HHV-8-infected individuals without KS disease, salivary DNA samples were first submitted to real-time PCR (q-PCR) to detect and quantify a 68-bp fragment of the HHV-8 genome which encodes for latent nuclear protein (ORF 73), as described by Krishnan et al.^[37]^ The limit of detection based on a standard curve was 4 copies of pGEX-5X plasmid containing the ORF 73 insert (donated by Harutaka Katano, Institute of Infectious Diseases, Tokyo, Japan).

### 2.4 Amplification of ORF K1, VR1 and VR2 sequences

We have analyzed the ORF K1 sequences to determine HHV-8 genotypes, sub-genotypes, and recombinant forms. Semi-nested PCR was performed by using Taq Platinum (Invitrogen Life Technologies, Carlsbad, CA) to obtain a complete ORF K1 sequence (867 bp), with primers and cycling parameters being adapted according to Lacoste et al^[36]^ and Poole et al.^[38]^ In parallel, the samples were also submitted to nested PCR to amplify shorter fragments of the ORF K1 hypervariable regions VR1 (386 bp) and VR2 (375 bp), with primers and cycling parameters being adapted according to Stebbing et al^[35]^ and Nascimento et al^[12]^ (Table 1). We used primers to amplify 3 different DNA fragments in order to evaluate the bias in HHV-8 DNA amplification and genotyping.

### 2.5 Nucleotide sequencing

PCR products were purified by using the QIAquick PCR Purification Kit (QIAGEN) and quantified with low DNA Mass ladder (Gibco). Approximately 20 ng of purified PCR were sequenced with Big Dye™ Terminator v 3.1 Cycle Sequencing Ready Reaction kit (Applied Biosystems, Foster City, CA) according to the manufacturer's instructions and using the inner primers of the nested PCR described in Table 1.

### 2.6 HHV-8 K1 genome and phylogenetic analysis

Fifty-two HHV-8 K1 sequences collected worldwide were obtained from GenBank (http://www.ncbi.nlm.nih.gov/) and used as references for analysis. References and complete K1 sequences were aligned by using MUSCLE software^[39]^ and manually edited with Se-Al (available at http://tree.bio.ed.ac.uk/software/sea/). Phylogenetic inference was performed under maximum likelihood (ML) by using RAxML v. 7.4.8^[40]^ with general time reversible (GTR)+Γ model of rate heterogeneity and 500 bootstrap replicates.

#### 2.6.1 Partial sequences of HHV-8 K1 and evolutionary placement algorithm (EPA)

Partial sequences of HHV-8 K1 were included in the alignment described above by using MAFFT software and the final alignment was submitted to EPA.^[41,42]^

By using the best ML phylogenetic tree consisting of HHV-8/ORF K1 as a reference and implementing the EPA, we have estimated the phylogenetic relationships in our collection of HHV-8 VR1, VR2, and K1 genome fragments. EPA was also performed by using RAxML v.7.4.8 with the GTR+Γ model.

### 2.7 Serology assay

The relationship between HHV-8 genotypes and positivity test to detect anti-HHV-8 against latency-associated nuclear antigen (LANA) and lytic-phase antigen was investigated by indirect immunofluorescence assay (IFA) according to the method described by Lennette et al^[43]^ and Camera Pierrotti et al.^[44]^

### 2.8 Statistical analysis

Data were analyzed with GraphPad Prism 5.0 (GraphPad Software Inc, San Diego, CA) and SigmaStat Analysis System Software 3.5 (Systat Software, Richmond, CA). The Mann–Whitney rank sum test was used for comparisons of 2 groups of continuous variables, whereas Yates’ chi-square correlation and 2-tailed Fisher's exact test, when appropriate, were used for comparisons of categorical data. The Kruskal–Wallis test was used for comparisons of 3 groups of continuous variables. Differences were considered statistically significant when *P* values were <0.05.

## 3 Results

### 3.1 Clinical and epidemiological characteristics of non-KS and KS groups

By using q-PCR, the fragment of ORF73 of HHV-8 was detected in the saliva of 106/751 (14.1%) individuals of the non-KS group (from 4 copies to 6.0 × 10^6^ copies/μg of DNA). By using nested PCR, complete or partial ORFK1 were successfully amplified from saliva samples of 34/106 (32.1%) non-KS individuals and from PBMC samples of 33/37 (89.2%) KS individuals. The genotyping of HHV-8 was successfully performed in 100% of the samples where the HHV-8/DNA was amplified by nested PCR, totalizing 67 AIDS individuals screened. The clinical and epidemiological characteristics of 67 AIDS individuals of the non-KS and KS groups are presented in Table 2. Both HIV viral load and the time of HIV/AIDS diagnosis were statistically different between non-KS and KS group (Table 2).

**Table 2:**
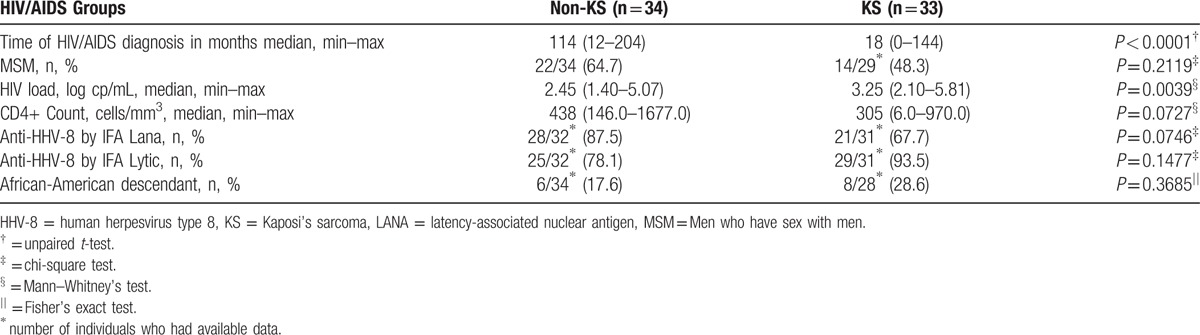
Clinical and epidemiological characteristics of non-KS and KS groups.

### 3.2 Genotype distribution and phylogenetic analysis

**Figure 1:**
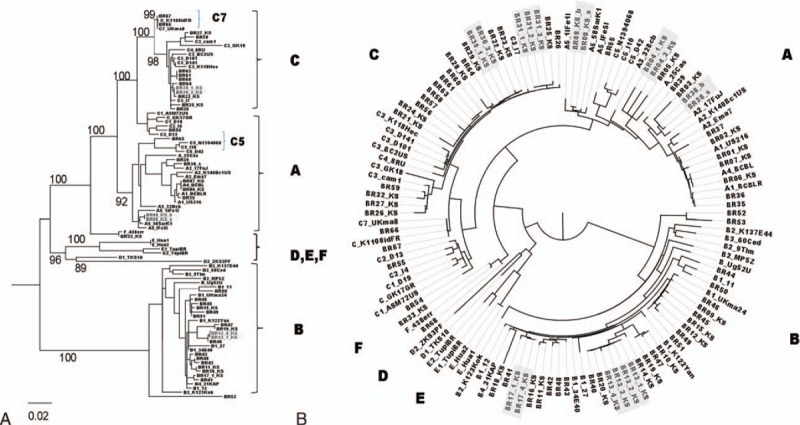
Phylogenetic tree. (A) HHV-8 K1 midpoint-rooted maximum likelihood (ML) tree based on the present study samples (bold) and the HHV8-K1 reference sequences using GTR + 4 Γ nucleotide substitution model. Support values after 1000 bootstrap runs are shown for each node. Only bootstrap values above 70 supporting each branch are shown. (B) Projection of the samples of HHV-8 VR1 and VR2 (bold) onto the scaffold of the HHV8 K1 ML tree using the evolutionary placement algorithm approach. There were 52 HHV-8 K1 sequences from other worldwide studies (GenBank references: http://www.ncbi.nlm.nih.gov/): AF178794 (25Cas), AF133038 (BCBLR), FJ884626 (US216), AF130305 (Ema7), AF178786 (17Fuj), AF178807(K1-40/Bc1US), AF178799 (K1-32/Bcb), AF133039 (BCBLB), AF130282 (1IFe1I), AF130284 (IFe5I), AF178823 (58sar), AF133040 (31 KAP), AF130290 (Ug52U), AF130301 (UKma24), AF178782 (K1-11), AF178783 (K1-12), AF178791 (K1-22/Yan), AF178796 (K1-27), AF178801 (K1-34/E40), AF171056 (9/Tim), AF178792 (K1-23/Kok), AF178787 (K1-18Hec), AF178804 (K1-37/E44), AY042940 (MP5Z), AF178825 (K1-60/Ced), AF278843 (10/Bid/J21), AF130274 (cam1), AF201851 (8RU), N1394068, AF130267 (G17), DQ394055 (D19), AF133041(ASM72US), DQ394048 (D13), DQ394058 (I4), AF133042 (BC2US), DQ394044 (D10-1), DQ394049 (D14-1), DQ394060 (I7), AF148805 (GK18), DQ394038 (D4-2), DQ394064 (I10), AF130304 (UKma8), AF278846 (TKS10), AF133044 (ZKS3PF), AY329027 (HUA1), AY329028 (HUA2), AF220292 (Tupi1BR), AF220293 (Tupi2BR) and AF178810 (K1-43/Berr). Taxa plus “BR” indicate the code of nucleotide sequences from this study. Codes BR36 to BR68 indicate nucleotide sequence from the non-KS group, and codes BR01_KS to BR33_KS indicate nucleotide sequences from the KS group. Taxa in gray area indicate HHV-8 sequences obtained from different blood samples or matched blood (indicated by “_b”) and saliva (indicated by “_s”) for the same individual. HHV-8 = human herpesvirus type 8, KS = Kaposi's sarcoma, ML = maximum likelihood.

Figure 1 depicts the HHV-8 phylogenetic reconstruction of partial and complete K1 sequences corresponding to 67 HIV-infected individuals without (n = 34) or with KS (n = 33). In the non-KS group, the proportions for genotypes A, B and C were 15.1%, 42.4%, and 42.4%, respectively. In the KS group, the proportions for genotypes A, B, and C were 25%, 37.5%, and 37.5%, respectively. By comparing the genotypic distribution of HHV-8 between non-KS and KS groups, no statistical difference was observed (Chi-square test, *P* = 0.6111). However, there was a statistical difference in relation to the distribution of genotypes A, B, and C within the non-KS group (*P* = 0.0252, Chi-square test). In the KS group, we did not find any statistical difference in distribution of genotypes. One sample from each group had virus from genotype F.

Matched saliva and blood samples from the same patient presented HHV8 DNA with close similarity to each other, as showed in the taxa BR38_b and BR38_s as well as BR08_KS_b and BR08_KS_s. In the KS group, 5 patients with serial blood samples available for analysis presented HHV-8 sequences with close similarity to their own earlier samples (Fig. 1B, gray area).

According to the Fig. 1A, 2 taxa (BR66 and BR67) clustered at positions intermediate to the sub-genotypes C1 and C2/C3 (i.e., intratype C7), and 1 taxon (BR65) between A3 and C3 (i.e., intertype C5).

There was no statistical difference in the genotypic distribution of HHV-8 among patients tested positive for anti-HHV-8 antibody against latency-associated nuclear antigen and lytic antigen by IFA in non-KS (Chi-square test, *P* = 0.8025) as well as KS (chi-square test, *P* = 0.9631) groups.

No statistical difference was observed in the genotypic distribution of HHV-8 between African and non-African ethnicities in non-KS (chi-square, *P* = 0.8811) as well as in KS (chi-square, *P* = 0.2935) groups. There was neither difference in the genotypic distribution of HHV-8 between visceral and non-visceral forms of disease in the KS group (chi-square test, *P* = 0.8259), nor HHV-8 viral load among HHH-8 genotypes in the non-KS group (Kruskal–Wallis test, *P* = 0.7126).

Table 3 (non-KS) and 4 (KS group) summarize the data corresponding to 67 HIV/AIDS-individuals whose HHV-8 genotypes A, B, C, and F were determined through VR1, VR2, or K1 sequences.

**Table 3:**
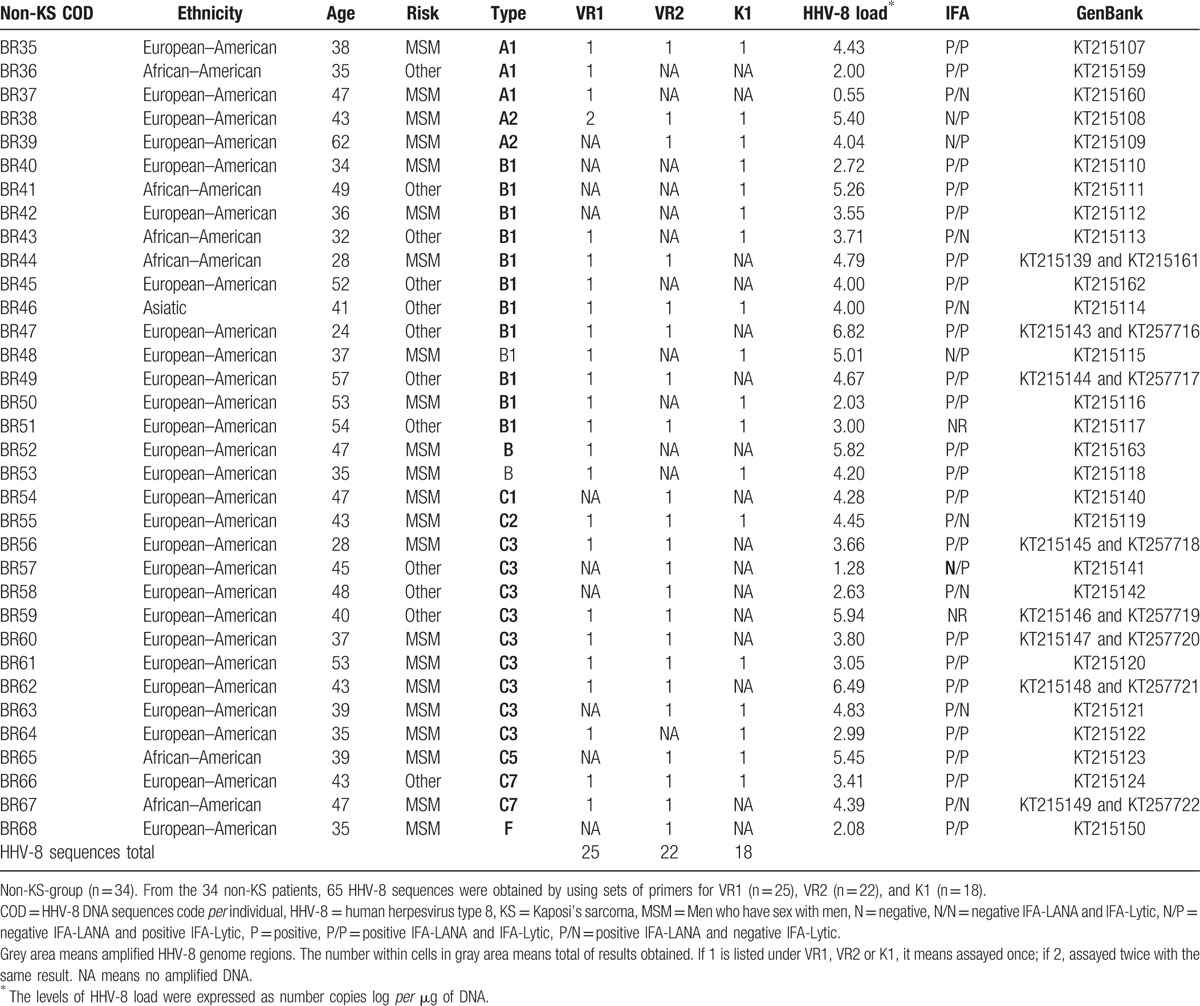
Non-KS group.

**Table 4:**
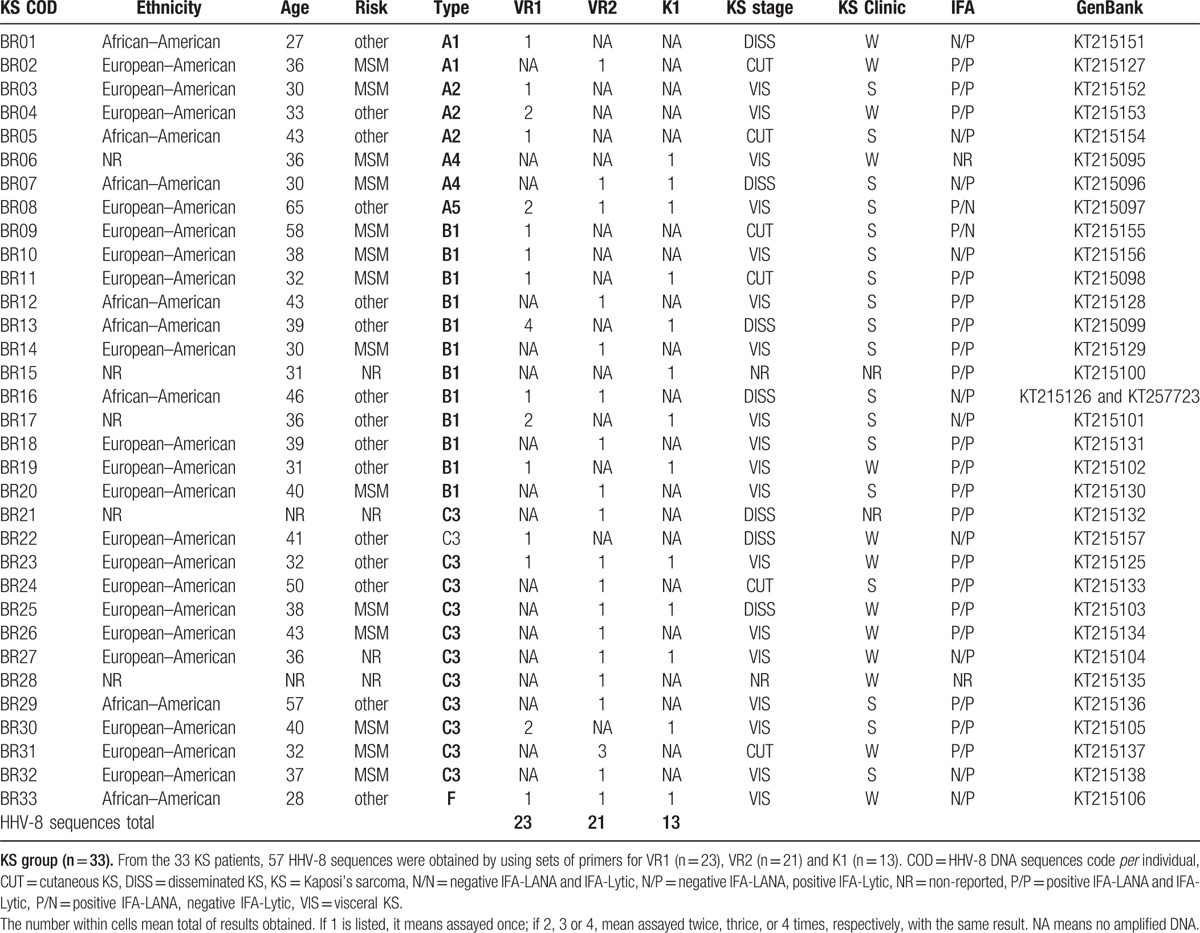
KS group.

### 3.3 Reducing biased HHV-8 genotyping

Most AIDS patients (n = 65) were distributed among the 3 different genotypes of HHV-8 (A, n = 13), (B, n = 26) and (C, n = 26) according to the genotyping assays by using the 3 sets of primes together (VR1, VR2 and K1), as showed in Tables 3–5. As showed in Table 5, VR2 assay was better to identify genotype C in comparison to A and B (*P* = 0.0001). VR2 assay did not amplify 16 of the 26 genotype B samples (61.5%) (*P* = 0.0005), whereas VR1 assay did not amplify 14 of the 26 genotype C samples (53.9%) (*P* = 0.0125). It was noticeable that certain primers preferentially amplify one genotype over another.

**Table 5:**
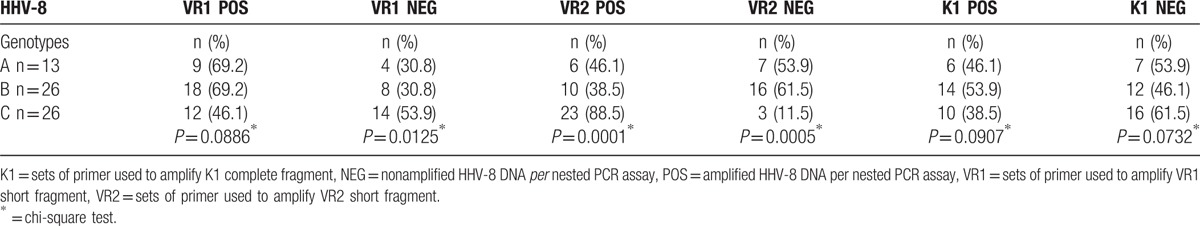
Distribution of HHV-8 A, B, and C genotypes according to PCR assays.

### 3.4 Prognosis of KS tumor according to HHV- 8 genotypes

The distribution of HHV-8 genotypes A, B, and C between subgroups W and S of the AIDS-KS group is presented in Table 6. It is possible to observe a better prognosis of KS in genotype B in comparison to other genotypes (Fisher's exact test, *P* = 0.0182). Genotype A in comparison to genotype non-A (Fisher's exact test, *P* = 0.6779) and genotype C in comparison to genotype non-C (Fisher's exact test, *P* = 0.0626) did not show any statistical difference regarding subgroups W and S.

**Table 6:**
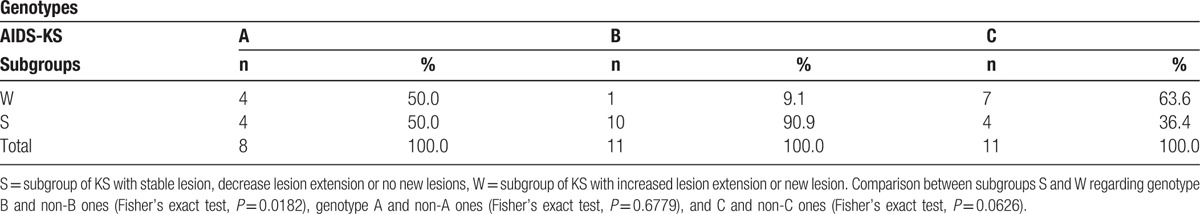
Distribution of genotypes A, B, and C between subgroups W and S in the KS group.

## 4 Discussion

We analyzed genetic variation of the HHV-8 K1 gene of a series of 67 viral isolates obtained from Brazilian individuals with AIDS. We also provide basic epidemiological information on the dissemination of HHV-8 genotypes, subgenotypes and recombinant forms for individuals with AIDS without KS and those with AIDS-KS.

Saliva proved to be a particularly convenient source for virus detection in asymptomatic HHV-8 infection, despite intermittent viral salivary shedding and low HHV-8 load in absence of the KS tumor.^[25,27,45,46]^ Nevertheless, prevalence of detectable HHV-8 salivary shedding in the group without KS was 14.1% (as assessed herein by means of real-time PCR from ORF 73), which is close to the prevalence rates reported in previous studies, that is, ranging from 9.5 to 11%.^[45,46]^ In the saliva samples from those non-KS individuals, our protocol using 3 sets of primers together (i.e., VR1, VR2, and K1) allowed us to genotype 32.1% of the samples, at even low viral load. In the PBMC samples from individuals with KS disease, our genotyping protocol reached 89.2% (33/37). The sensitivity reported in previous studies was lower compared to the present one, with estimates varying from 26% to 74% in the analysis of 1 or both HHV-8 ORF-K1 partial targets in biopsy specimens of AIDS-KS lesions.^[12,47]^

Our comparative data on the genotyping performance show that VR2 assay resulted in underestimation of HHV-8 genotype B, but worked very well to genotype C. This might be attributed to the fact that certain primers preferentially amplify one genotype over another. In previous studies conducted in South America, HHV-8 genotype B prevalence ranged from 2% to 21%.^[12,20]^ The discordant data on the prevalence of genotype B among febrile children in Africa was also observed.^[17,22]^ Discrepancies among genotype B frequencies partly reflect the need to achieve a standard method for ORF K1 amplification in genotyping studies, thus allowing for substantial detection of HHV-8 variants. Analysis of the alignment containing reference sequences of HHV-8 revealed important mismatches within the sites of the primers set for VR1 and VR2 (data not shown), which ultimately may lead to the biased genotyping. Based on our genotyping protocol, we could reduce biased HHV-8 genotyping by using the 3 PCR assays together (VR1, VR2, and complete K1).

We have found genotypes, sub-genotypes and recombinant forms of HHV-8 similar to those described worldwide (Fig. 1). It is noticeable a predominance of European-American descendants (77.0%), which reflects the predominantly European ancestry of São Paulo patients, with HHV-8 genotypes A, B, C, and F. For the first time we have described the occurrence of genotype F in Brazil, which had been previously described in Bantu tribe of Sub-Saharan Africa.^[26]^ Unlike previous studies demonstrating a high prevalence of HHV-8 genotype A among individuals with AIDS-KS in the United States, Latin America, Northern Europe and Asia,^[12,20,21,23,24,48]^ we have observed a lower prevalence of genotype A in relation to genotypes B and C in both non-KS and KS groups in AIDS individuals, reaching statistical significance particularly in the former group. In fact, our data regarding the predominance of genotypes B and C in Brazilian HHV-8 isolates agree with previous report by Caterino^[18]^ which was based on a small fragment of the ORF-26 gene.

With regard to the phylogenetic analysis, the genetic diversity present in the clade C of HHV-8 (C1, C2, C3, or C7) from non-KS patients was similar to that previously reported in a HHV-8 endemic African country,^[49]^ including slowly progressing classic KS and intra-familial transmission in the Mediterranean countries.^[8,14,50,51]^ On the other hand, there was a lack of diversity within clade C in the KS group, which was composed of 100% of HHV-8 subgenotype C3, (Tables 3 and 4). Moreover, sequencing the complete ORF K1 allowed us to identify recombinant forms previously described as C5 and C7 in the saliva samples from non-KS individuals (Fig. 1A). Our data corroborate the previous study showing evidence of recombinant virus patterns C5 (A3 and C3) and C7 (C1 and C2/C3), which were described in classic and epidemic KS.^[49]^

Although recombinant forms of HHV-8 were found in non-KS group, we did not find co-infection with different HHV-8 genotypes in any patient analyzed, including 7 cases for which serial blood or matched blood and saliva were sampled in time interval up to 8 years (Fig. 1B, gray area). In fact, most studies consistently demonstrate that co-infection by different HHV-8 genotypes is extremely rare,^[26,46,50–52]^ thus supporting that the currently detected recombinant forms do not originate from new events, but they represent previous circulating recombinant forms.^[2,49]^

The relationship between the clinical progression of KS and HHV-8 genotype is still unclear.^[2,7,8,10,12,13]^ As far as we are aware, this study is the first to show the predominance of HHV-8 genotype B among individuals with AIDS-KS presenting reduced lesion extension or no new lesions (subgroup S) (Table 6). Although some previous reports on the clinical progression of KS have demonstrated an associated between HHV-8 genotype A and fast progressing endemic and classic KS patients,^[7,8,10]^ other studies have not led to the same results.^[2,12,13]^

Our study, however, has some limitations. First, it was not possible to obtain serially CD4 count and HIV viral load, so it would be very useful to verify some additional relationship with clinical progression of KS. Second, the limited number of samples analyzed precludes detailed investigation on the impact of ethnicity on HHV-8 genotypes.

## 5 Conclusion

In conclusion, the distribution of HHV-8 genotypes A, B, C, and F in AIDS individuals was indistinguishable between the groups with and without KS. However, it was possible to suggest that genotype B might be associated with better tumor prognosis in the KS group. Interestingly, salivary shedding of HHV-8 among AIDS individuals without KS presented a particular diversity of strains within clade C and 2 already described recombinant patterns. Moreover, we emphasize the need to achieve a standard method for ORF K1 amplification in genotyping studies, thus allowing for substantial detection of HHV-8 variants.

## Acknowledgments

The authors thank Dr. Vanda Akiko Ueda Fick de Souza (Institute of Tropical Medicine Laboratory of Virology, São Paulo, Brazil) for both helpful insights and support to this study as well as LIM 52 (HCFM-USP) and *FAPESP* (State of São Paulo Research Support Foundation) for funding resources.

CNPq (National Counsel of Technological and Scientific Development, number 300944/2013-6).
