# Genotypic distribution of HHV-8 in AIDS individuals without and with Kaposi sarcoma: Is genotype B associated with better prognosis of AIDS-KS?: Erratum

**DOI:** 10.1097/MD.0000000000006194

**Published:** 2017-02-17

In the article “Genotypic distribution of HHV-8 in AIDS individuals without and with Kaposi sarcoma: Is genotype B associated with better prognosis of AIDS-KS?”,^[1]^ which appeared in Volume 95, Issue 48 of *Medicine*, Dr. de Oliveira's first name was originally misspelled. Her first name is Cristina.

## References

[1] Tozetto-Mendoza TR Ibrahim KY Tateno AF Genotypic distribution of HHV-8 in AIDS individuals without and with Kaposi sarcoma: Is genotype B associated with better prognosis of AIDS-KS? *Medicine*. 2016 95:e5291. 27902590 10.1097/MD.0000000000005291 PMC5134807

