# Genotypic distribution of HHV-8 in AIDS individuals without and with Kaposi sarcoma: Is genotype B associated with better prognosis of AIDS-KS?: Erratum

**DOI:** 10.1097/MD.0000000000006992

**Published:** 2017-05-19

In the article, “Genotypic distribution of HHV-8 in AIDS individuals without and with Kaposi sarcoma: Is genotype B associated with better prognosis of AIDS-KS?”,^[1]^ which appeared in Volume 95, Issue 48 of *Medicine*, Dr. Cristina Mendes de Oliveira's name appeared incorrectly as “Cristine Mendes de Oliveira.”
